# Chromosome-level assembly of *Gymnocypris eckloni* genome

**DOI:** 10.1038/s41597-022-01595-w

**Published:** 2022-08-02

**Authors:** Fayan Wang, Lihan Wang, Dan Liu, Qiang Gao, Miaomiao Nie, Shihai Zhu, Yan Chao, Chaojie Yang, Cunfang Zhang, Rigui Yi, Weilin Ni, Fei Tian, Kai Zhao, Delin Qi

**Affiliations:** 1 State Key Laboratory of Plateau Ecology and Agriculture, Qinghai University, Xining, 810016 China; 2 College of Eco-Environmental Engineering, Qinghai University, Xining, 810016 China; 3 Animal Science Department of Agriculture and Animal Husbandry College, Qinghai University, Xining, 810016 China; 4 Key Laboratory of Adaptation and Evolution of Plateau Biota, Northwest Institute of Plateau Biology, Chinese Academy of Sciences, Xining, 810001 China

**Keywords:** Genome, Ichthyology

## Abstract

*Gymnocypris eckloni* is widely distributed in isolated lakes and the upper reaches of the Yellow River and play significant roles in the trophic web of freshwater communities. In this study, we generated a chromosome-level genome of *G. eckloni* using PacBio, Illumina and Hi-C sequencing data. The genome consists of 23 pseudo-chromosomes that contain 918.68 Mb of sequence, with a scaffold N50 length of 43.54 Mb. In total, 23,157 genes were annotated, representing 94.80% of the total predicted protein-coding genes. The phylogenetic analysis showed that *G. eckloni* was most closely related to *C. carpio* with an estimated divergence time of ~34.8 million years ago. For *G. eckloni*, we identified a high-quality genome at the chromosome level. This genome will serve as a valuable genomic resource for future research on the evolution and ecology of the schizothoracine fish in the Qinghai-Tibetan Plateau.

## Abstract (summary)

**Table:** 

Measurement(s)	Genome
Technology Type(s)	Whole Genome Sequencing
Sample Characteristic - Organism	Gymnocypris eckloni
Sample Characteristic - Environment	fresh water
Sample Characteristic - Location	Little Yellow River

## Background & Summary

The Qinghai-Tibetan Plateau (QTP) is the highest and one of the biggest plateaus on earth, covering 2.5 × 10^6^ square kilometers with an elevation of 3000–5000 m for most parts of the area. The intensive uplifts of QTP resulted from collision of the India plate and the Eurasia plate had a profound impact on the climate and environment^1,2^. Characterized by high altitude, low oxygen partial pressure (hypoxia), low temperatures, dramatic temperature fluctuations, and high UV radiation, the QTP environment posed harsh challenges to the endemic animals^3,4^. Recently, comparative genomic studies of animals endemic to the QTP provide valuable clues for scientists to understand the molecular mechanism of environmental adaptation^4–8^. However, the genome information of fish species in QTP is still lacking.

Schizothoracine fish (Teleostei: Cyprinidae) are the largest and most diverse taxon within the QTP ichthyofauna and their radiation has been correlated with the plateau’s rapid upheaval^9,10^. The schizothoracine fish, confined to regions at either high altitudes or high latitudes, have evolved a number of unique traits (i.e., degeneration of body scales, slow growth, and late sexual maturity) that adapt to the extreme environment of the QTP and play significant roles in the trophic web of QTP freshwater communities^10–13^. Therefore, the schizothoracine fish have been accepted as ideal models for studying the molecular mechanisms underlying the adaptation to harsh environments^11–13^.

The schizothoracine fish comprises 11 or 12 genera and approximately 100 species and are mainly distributed in cold tributaries and lakes of the QTP and adjacent areas at 2000 m above sea level^10,11^. The phylogenetic analysis based on morphological traits revealed that the schizothoracine fishes can be divided into three sub-groups including primitive, specialized and highly specialized group^10^, which was proposed to be associated with the tectonic upshifts of the QTP^14–16^. Previous studies have shown that the karyotypes of the schizothoracine fish range from 90 to 446 and that almost all species were polyploid^17–20^. A recent genomic study confirmed that *Schizothorax o’connori* of Schizothoracinae was a young tetraploid that underwent a fourth whole-genome duplication (4 R WGD) after the teleost-specific third WGD (3 R WGD)^21^. Other studies indicated that the globin gene superfamily, toll-like receptor family, and interferon regulatory factors in a representative species from this subfamily underwent adaptive evolution in response to the plateau environment, specifically gene loss, and gain events as a result of genome and/or gene duplications^13,22–24^. *Gymnocypris eckloni* is a representative species of the highly specialized schizothoracine fish that is widely distributed in isolated lakes and the upper reaches of the Yellow River, and is very well adapted to the plateau’s aqueous environment^9,10^. Investigating the genomic evolution of *G. eckloni* may shed light on the underlying molecular mechanisms involved in high-altitude adaptations in schizothoracine fish of the QTP.

In the present study, we integrated PacBio long-read sequencing, Illumina short-read sequencing, and high-throughput chromosome conformation capture (Hi-C) technology to generate a high-quality chromosome-level reference genome for *G. eckloni*. The reference genome obtained in this study will provide a foundation for future investigations on the evolution and adaptation of schizothoracine fish.

## Methods

### Experimental fish and sequencing

*G. eckloni* genomic DNA were extracted from the muscle samples of a healthy female individuals obtained from the Native Fish Artificial Proliferation and Release Station, Xunhua, Qinghai Province, China (Fig. s1). For genome assembly, two libraries with insert sizes of 300 bp and 20 kb were separately constructed using an Illumina TruSeq Nano DNA Library Prep Kit and SMRT bell Template Prep Kit. The two libraries were subsequently sequenced using an Illumina HiSeq X Ten instrument and a PacBio Sequel platform^25^. For the PacBio platform, a total of 312.2 Gb PacBio long sequencing reads were generated, and 239.0 Gb subreads (334.6 × coverage) with an average length of 23,706 bp were obtained after removing adaptors in polymerase reads (Table 1). For the Illumina HiSeq X Ten sequencing platform, a total of 251.7 Gb short sequencing reads were generated. After filtering, 215.2 Gb (231.2 × coverage) of clean Illumina data were retained to perform a genome survey.

**Table 1:** Sequencing data used for the genome *G. eckloni* assembly.

Library types	Insert size (bp)	Raw data (Gb)	Clean data (Gb)	Read length (bp)	Sequence coverage (X)
Illumina reads	300	215.7	215.2	150	231.2
PacBio reads	20000	312.2	239.0	23706	334.6
Hi-C reads	—	257.3	257.3	300	275.8
RNA reads	300	67.76	66.43	150	—
Total	—	852.96	777.93	—	—

To conduct chromosome-level assembly of the *G. eckloni* genome, a Hi-C library was generated using the *Mbo* I restriction enzyme following previously described standard protocol with minor modifications^26^. In brief, the purified DNA from the fresh muscle sample was digested with Mbo I restriction enzyme and labelled by incubating with Biotin-14-dATP (Thermo Fisher Scientific, USA), and then ligated by T4 DNA Ligase. After incubating overnight to reverse crosslinks, the ligated DNA was sheared into 200–600 bp fragments, and then blunt-end repaired and A-tailed, followed by purification through biotin-streptavidin-mediated pull down. Finally, the Hi-C libraries were quantified and sequenced on the Illumina NovaSeq6000 platform (Illumina, USA) using a PE-150 module, generating a total of 257.3 Gb (275.8 × coverage) clean data after using the same filter criteria with short reads (Table 1).

To provide evidence of transcripts for genome structure annotation, we conducted RNA-seq for muscle, skin, gill, liver, gut, spleen, kidney, heart, eye and blood samples. RNA was extracted using Ambion MagMAX-96 total RNA isolation kit (Life Sciences, United States) for all samples, and DNase I treatment was performed to eliminate DNA contamination. After the quality assessment of the extracted RNAs using NanoPhotometer® spectrophotometer (Implen, United States), RNA-seq libraries were constructed according to the protoco and were sequenced by Illumina HiSeq4000 in paired-end 150 bp mode, resulting in a total of 66.43 Gb clean transcriptome data (Table 1).

### *De novo* assembly of *G. eckloni* genome

We used the k-mer method to survey the genomic features of the *G. eckloni*. The k-mer count histogram was obtained from Illumina paired-end sequencing data using Jellyfish v2.99^27^. Based on the total number of 169,021,371,761 17-mers and a peak 17-mer depth of 181, the genome size of *G. eckloni* was estimated to be 927.13 Mb, and the estimated heterozygosity rate was approximately 1.82% (Table s1).

The 239.0 Gb subreads from the PacBio Sequel platform were used for genome assembly using wtdbg2^28^ followed by Quiver^29^ and Pilon^30^ polishing using the 215.2 Gb of Illumina HiSeq clean reads, which produced a 918.45 Mb genome assembly, consisting of 3,170 contigs with a contig N50 size of 4.19 Mb (Table 2).

**Table 2:** The statistics of length and number for the de novo assembled *G. eckloni* genome.

Term	Length		No.	
	Contig (bp)	Scaffold (bp)	Contig	Scaffold
**Total**	918,450,624	918,681,488	3,170	711
**Max**	22,682,260	89,391,071	—	—
**Number > = 2000**	—	—	3,058	711
**N50**	4,192,824	43,543,958	56	8
**N60**	2,476,204	34,715,927	85	11
**N70**	1,500,513	32,896,108	133	13
**N80**	641,416	29,129,546	229	16
**N90**	146,685	25,669,045	553	20

**Fig. 1:**
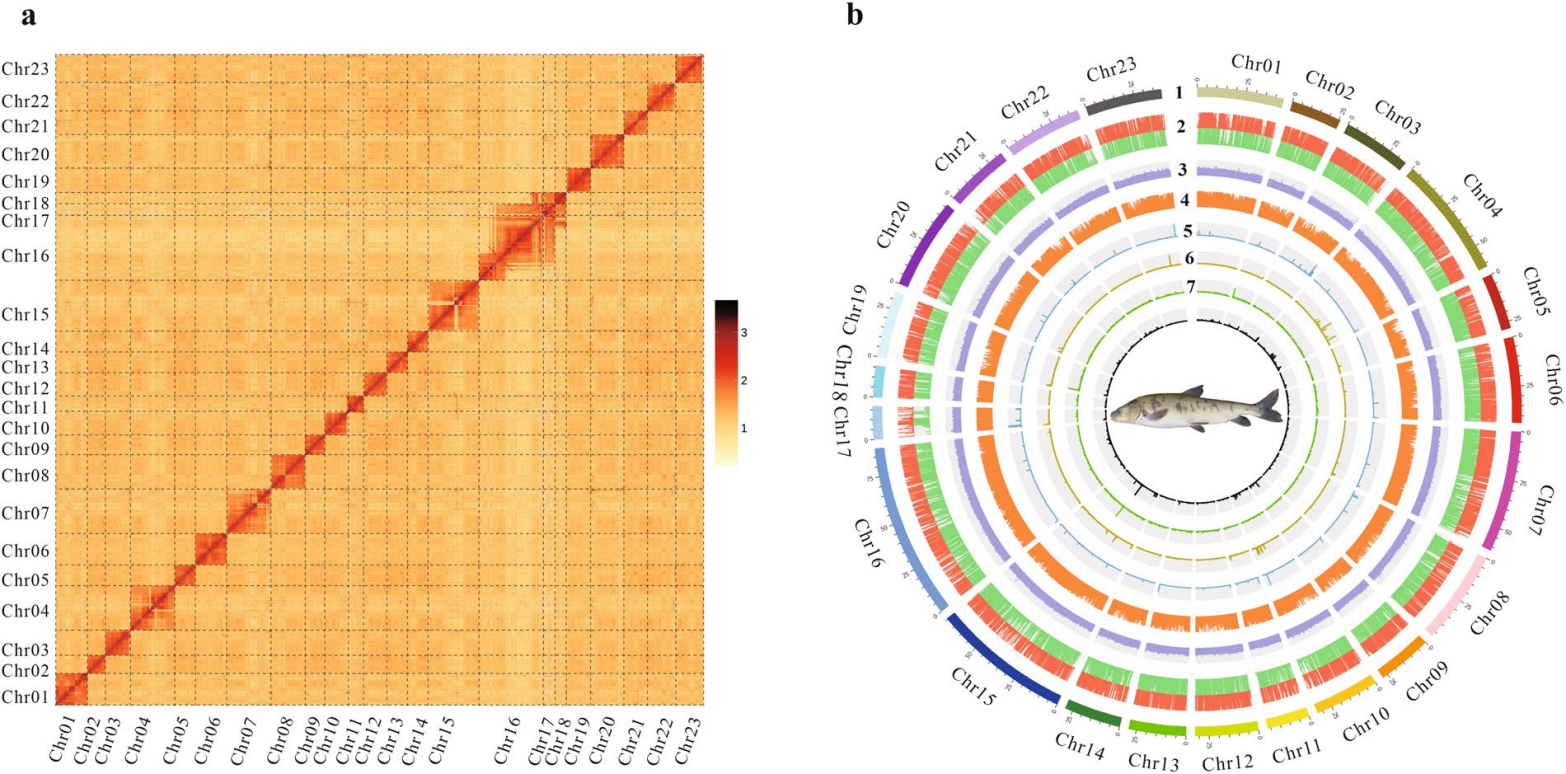
Characteristics of the *G. eckloni* genome. (**a**) Hi-C intra-chromosomal contact map of the *G. eckloni* genome assembly. (**b**) Circos plot of the *G. eckloni* genome assembly. 1) Pseudo-chromosomes; 2) gene distribution; 3) GC content; 4) repeat distribution; 5) rRNA distribution; 6) tRNA distribution; 7) miRNA distribution; 8) snRNA distribution. All data were obtained using a sliding window of 10 Kb.

Hi-C technology was applied to conduct the chromosome-level genome assembly of *G. eckloni*. Clean reads sequenced from the Hi-C library were aligned to the contig-level genome with an end-to-end algorithm implemented in Bowtie v2.3.5 according to the Hi-C-Pro strategy^31,32^. Juicer v1.6.2 and 3D *de novo* assembly (3D-DNA) pipelines were used to assemble the contigs into the chromosome-level genome^33,34^. Ultimately, the assembled sequences were further anchored and orientated onto 23 pseudo-chromosomes using Hi-C data. The 23 pseudo-chromosomes ranged in size from 15.91 to 89.39 Mb (Fig. 1 and Table s2), covering ~98.52% of the whole genome. Finally, the *G. eckloni* genome was obtained with 711 scaffolds and a total length of 918,681,488 bp, a contig N50 of 4.19 Mb, and scaffold N50 of 43.54 Mb (Table 2).

The completeness of the genome assembly was assessed by the single copy orthologs (BUSCO, version 5.3.2)^35^ and CEGMA^36^ software. The BUSCO analysis based on the actinopterygii_odb10 database showed that 87.5% (single-copy genes: 83.0%, duplicated genes: 4.5%) of the 3,640 single-copy genes were identified as complete, 1.3% were fragmented, and 11.2% were missing from the assembled genome. The CEGMA analysis revealed that 221 conserved genes (89.11% of the core eukaryotic genes) supported the completeness of the assembled genome. Illumina short reads were mapped to the assembled genome using BWA^37^ software to evaluate completeness of the genome assembly. The results showed that 93.40% of the reads could be mapped, covering 96.34% of the assembled genome.

### Repetitive element and non-coding gene annotation in the *G. eckloni* genome

A combined strategy using homology alignments and *de novo* searches to identify whole-genome repeats was applied in our repeat annotation pipeline. Tandem repeats were extracted using TRF (http://tandem.bu.edu/trf/trf.html) by *ab initio* prediction. For homolog prediction, Repbase (http://www.girinst.org /repbase) employing RepeatMasker (http://www.repeatmasker.org/) software and its in-house scripts (RepeatProteinMask) with default parameters was used to extract repeat regions. Additionally, *ab initio* prediction based on the *de novo* repetitive elements database was conducted by LTR_FINDER (http://tlife.fudan.edu.cn/ltr_finder/), RepeatScout (http://www.repeatmasker.org/), and RepeatModeler (http://www.repeatmasker.org/RepeatModeler.html) with default parameters. Then, all repeat sequences with lengths > 100 bp and gap ‘N’ < 5% were used to construct the raw transposable element (TE) library. A custom library (a combination of Repbase and our *de novo* TE library, which was processed by uclust to yield a non-redundant library) was supplied to RepeatMasker for DNA-level repeat identification. The results showed revealed that 47.63% of the *G. eckloni* genome was annotated as repetitive elements (Table s3), of which LTRs were the most abundant with a total length of 356.79 Mb, accounting for 38.84% of the whole genome. SINEs were the rarest with a total length of 2.37 Mb and represented 0.26% of the whole genome (Table s4).

The tRNAs were predicted using tRNAscan-SE (http://lowelab.ucsc.edu/tRNAscan-SE/), and the rRNA sequences were predicted using BLAST. The results showed that a total of 12,157 tRNAs were predicted using tRNAscan-SE, and 1,780 rRNA genes were annotated using BLASTN tool with an E-value of 1E-10^32^ against human rRNA sequence. Other ncRNAs, including miRNAs and snRNAs, were identified by searching against the Rfam database with default parameters using infernal software (http://infernal.janelia.org/) (Table s5).

### Annotation of protein-coding genes

**Fig. 2:**
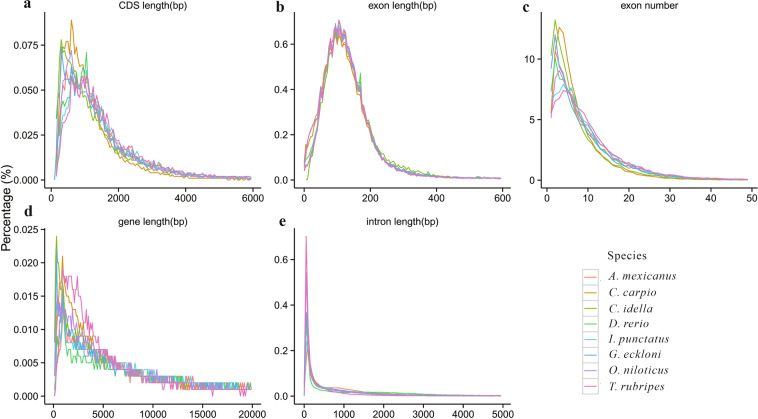
The composition of gene elements in the *G. eckloni* genome to other species. (**a**) CDS length distribution and comparison with other species. (**b**) Exon length distribution and comparison with other species. (**c**) Exon number distribution and comparison with other species. (**d**) Gene length distribution and comparison with other species. (**e**) Intron length distribution and comparison with other species.

Gene predictions were conducted through a combination of homology, *de novo*, and transcriptome-based prediction methods. For homology-based predictions, the protein sequences of seven fish species, including *Oryzias latipes*, *Ctenopharyngodon idellus*, *Ictalurus punctatus*, *Cyprinus carpio*, *Takifugu rubripes*, *Danio rerio*, and *Astyanax mexicanus*, were downloaded from Ensembl database (http://asia.ensembl.org/index. html). Protein sequences were aligned to the genome using TblastN v2.2.26 with an e-value of 1e^−5 ^^38^. Then, matching proteins were aligned to homologous genome sequences for accurate spliced alignments using GeneWise v2.4.1^39^ (referred to “Homolog” in Table 3), which was subsequently used to predict gene structure of each protein region. RNA-sequencing data derived from nine tissues and blood samples were assembled using Trinity v2.1.1^40^, and were aligned against the *G. eckloni* genome using Program to Assemble Spliced Alignment (PASA)^41^ (referred to “PASA” in Table 3). To optimize genome annotation, RNA-seq reads from different tissues were aligned to *G. eckloni* genome fasta using TopHat package v2.0.11 with default parameters to identify exons region and splice positions^42^. The alignment results were then used as inputs for Cufflinks package v2.2.1 with default parameters for genome-based transcript assembly^43^ (referred to “Cufflinks”in Table 3). Finally, EvidenceModeler v1.1.1 was used to combine the gene models into weighted consensus gene structures with masked repetitive elements^41^. Additionally, PASA was used to update the final gene models, thereby adding information of alternatively spliced sites and untranslated regions (UTR) (referred to “Pasa-update” in Table 3). Ultimately, a total of 24,430 protein-coding genes were predicted in the *G. eckloni* genome. The average transcript length was 16,219.34 bp with an average coding sequence (CDS) length of 1,536.71 bp. The average exon number per gene was 8.88 with an average exon length of 173.00 bp and average intron length of 1,862.69 bp (Table 3). The statistics of gene models, including lengths of a gene, CDS, intron, and exon in *G. eckloni* were comparable to those for close-related species (Table s6 and Fig. 2).

**Table 3:** Gene annotation of *G. eckloni* genome via three methods.

Method	Gene set	Number	Average length (bp)				Exons No. per gene
			Transcript	CDS	Exon	Intron	
De novo	Augustus	38,431	9,427.68	1,102.72	181.26	1,637.57	6.08
	GlimmerHMM	88,372	9,368.19	580.21	146.67	2,973.05	3.96
	SNAP	47,478	20,534.02	796.89	143.55	4,336.44	5.55
	Geneid	32,716	17,045.39	1,223.45	216.31	3,398.15	5.66
	Genscan	32,712	19,569.14	1,429.87	189.78	2,775.94	7.53
Homolog	*Oryzias latipes*	18,845	11,159.32	1,293.66	179.22	1,586.58	7.22
	*Ctenopharyngodon idellus*	24,602	9,475.91	1,264.07	184.13	1,400.12	6.87
	*Ictalurus punctatus*	19,535	13,585.62	1,522.10	182.89	1,647.47	8.32
	*Cyprinus carpio*	23,776	10,240.81	1,276.54	182.36	1,494.04	7.00
	*Takifugu rubripes*	18,028	13,503.98	1,497.61	181.74	1,658.25	8.24
	*Danio rerio*	20,929	13,270.63	1,510.71	180.45	1,595.28	8.37
	*Astyanax mexicanus*	20,090	11,862.97	1,393.39	185.72	1,610.03	7.50
RNAseq	PASA	91,220	14,128.66	1,240.08	165.12	1,979.72	7.51
	Transcripts	66,837	31,133.65	2,702.91	300.16	3,551.63	9.00
EVM		35,931	11,908.94	1,192.33	176.57	1,862.87	6.75
Pasa-update		35,599	12,447.20	1,220.20	177.47	1,910.77	6.88
Final set		24,430	16,219.34	1,536.71	173.00	1,862.69	8.88

Note that CDS refers to coding sequence; GlimmerHMM was a new genefinder based on a Generalized Hidden Markov Model (GHMM); SNAP refers to Semi-HMM-based Nucleic Acid Parser; EVM refers to Evidence modeler.

**Fig. 3:**
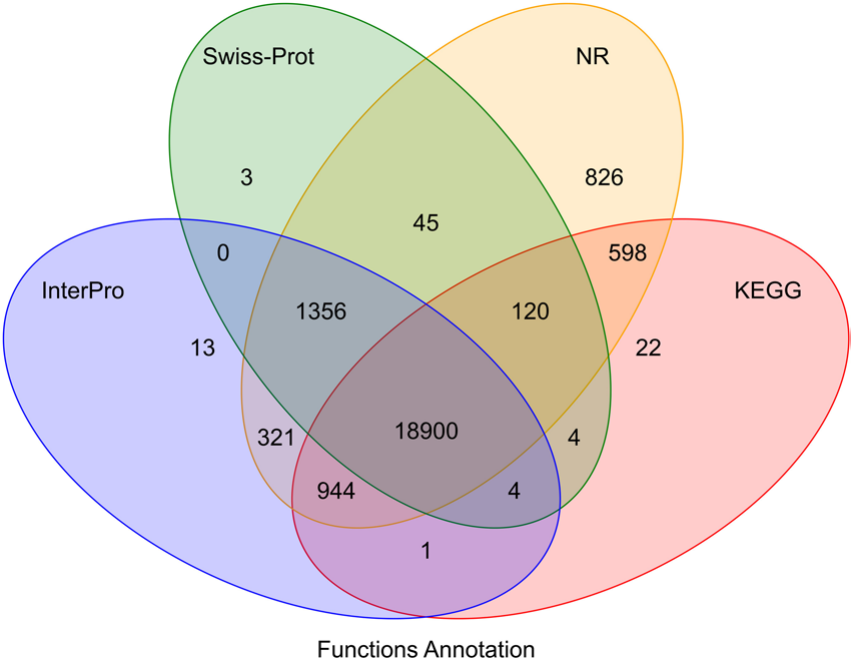
Venn diagram of number of genes with homology or functional classification by each method.

Public biological function databases of NR, SwissProt^44^, InterPro^45^, and Kyoto Encyclopedia of Genes and Genomes (KEGG) databases^46^ were used for the functional annotation of protein-coding genes using BLASTX and BLASTN utilities^46^ with an e-value threshold of 1e^−5^. InterPro database was used to predict protein function based on the conserved protein domains by InterproScan tool^47^. A total of 23,157 genes (94.8%) were successfully annotated by at least one public database (Table s7 and Fig. 3).

### Evolutionary and comparative genomic analysis

**Fig. 4:**
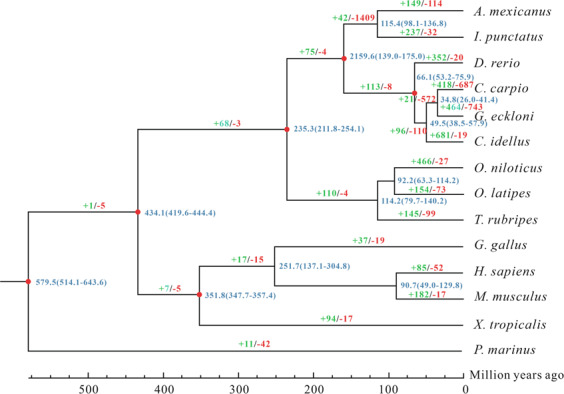
Phylogenetic tree based on single-copy genes from 14 species shows the estimated divergence time (blue numbers), topology and expansion (green numbers), and contraction (red numbers) of gene families.

To examine *G. eckloni* evolution, we used OthoMCL^48^ to cluster its genes with those from 13 other vertebrates: *Astyanax mexicanus, Ictalurus punctatus, Danio rerio, C. carpio, Ctenopharyngodon idella, Oreochromis niloticus, Oryzias latipes, Takifugu rubripes, Gallus gallus, Homo sapiens, Mus musculus, Xenopus tropicalis, and Petromyzon marinus*. From these 14 species, we identified 597 one-to-one single-copy genes that were used to construct a maximum likelihood (ML) tree using RaxML with the GTRGAMMA model^49^. Divergence times between species were calculated using the MCMC tree program implemented by PAML package^50^. According to the time-calibrated phylogeny, the age of the most recent common ancestor (MRCA) of the teleost fish was estimated to be 211.8–254.1 million years ago. The *G. eckloni* with the closest relationship to *C. carpio* shared an MRCA at ~ 34.8 million years ago (Fig. 4).

A total of 24,619 gene families were identified among the 14 species (Table s8), of which 2,739 core gene families were shared by all 14 species and 856 gene families is unique for *G. eckloni* including 1,488 genes. Analysis of the expansion and contraction of the gene families revealed that there were 464 (1650 genes) expanded and 743 (192 genes) contracted gene families in *G. eckloni* when compared to its MRCA (Fig. 4). The expanded gene families included ABC transporters, Peroxisome, Herpes simplex virus 1 infection, Staphylococcus aureus infection, Axon guidance, Dorso-ventral axis formation, Pertussis, Legionellosis, Rap1 signaling pathway and so on, and the contracted gene families included Tight junction, Systemic lupus erythematosus, Pathogenic Escherichia coli infection, Gap junction, Alcoholism, Pertussis, Ascorbate and aldarate metabolism, NOD-like receptor signaling pathway and so on.

## Data Records

All raw data of the whole genome have been deposited into the National Center for Biotechnology Information (NCBI) SRA database (Experiments for SRP377513) under BioProject accession number PRJNA835611^51^. The assembled genome has been deposited at DDBJ/ENA/GenBank under the accession JAMHKY000000000^52^. Data of the expansion and contraction of the gene families, gene functional annotations, repeat annotation and results of evolutionary analysis had been deposited at Figshare^53^.

## Technical Validation

### RNA integrity

The transcriptomes for nine tissues and blood from three fish individuals were sequenced. Before constructing RNA-Seq libraries, RNA purity was analyzed with a NanoPhotometer Spectrophotometer (Implen, United States). The RNA concentration was quantified with a Qubit RNA Assay Kit in a Qubit 2.0 Fluorometer (Life Technologies, United States). RNA integrity was analyzed using a RNA Nano 6000 Assay Kit and an Agilent Bioanalyzer 2100 (Agilent Technologies, United States). The total amount of RNA, RNA integrity and rRNA ratio were used to estimate the quality, content and degradation level of RNA samples. In the present study, RNAs samples with a total RNA amount ≥ 10 μg, RNA integrity number ≥ 8, and rRNA ratio ≥ 1.5 were finally subjected to construct the sequencing library.

### Comparative genomic analyses

The protein sequences of 13 vertebrates, including *A. mexicanus, I. punctatus, D. rerio, C. carpio, C. idella, O. niloticus, O. latipes, T. rubripes, G. gallus, H. sapiens, M. musculus, X. tropicalis, and P. marinus*, were downloaded from the Ensembl database (Release 98). Orthologous relationships between the genes from *G. eckloni* and the 13 other vertebrates were inferred through all-against-all protein sequence similarity searches using OthoMCL^48^. Only the longest predicted transcript per locus was retained. In the all-against-all BLASTP comparisons, a cutoff e-value of 1e^−5^ was used. The MCL inflation index was set to 1.5.

For each gene family, an alignment was produced using Muscle (http://www.drive5.com/muscle/), and ambiguously aligned positions were trimmed using Gblocks (http://molevol.cmima.csic.es/castresana/Gblocks.html). The tree was inferred using RAxML^49^. The best-scoring ML tree was inferred by a rapid bootstrap algorithm and ML searches after performing 1000 rapid bootstrap replications. Divergence times between species were calculated using the MCMC tree program implemented by PAML package^50^. The divergence times for *D. rerio* vs *C. idella* (48–75 Ma), *A. mexicanus* vs *C. carpio* (137–174 Ma), *C. carpio* vs *T. rubripes* (206–252 Ma), *G. gallus* vs *X. tropicalis* (347.6–358.3 Ma), *T. rubripes* vs *G. gallus* (413–443 Ma), and *G. gallus* vs *P. marinus* (515–646 Ma) were obtained from the TimeTree database then used to calibrate divergence dates of other nodes on the phylogenetic tree^54^.

According to the divergence times and phylogenetic relationships, CAFÉ was used to analyze the expansion and constriction of gene families in the *G. eckloni* genome based on the gene families identified by OrthoMCL^55^. The phylogenetic tree topology and branch lengths were taken into account when inferring the significance of change in the gene family size of each branch. Enrichment analyses based on the Gene Ontology (GO) and KEGG annotations were performed to identify the functional implications of expanded and contracted genes (Fisher’s exact test, adjusted *p*-value < 0.05).

## Supplementary information

Supplementary information of Chromosome-level assembly of Gymnocypris eckloni genome

## Data Availability

All software used in this work is in the public domain, with parameters being clearly described in Methods. If no detail parameters were mentioned for a software, default parameters were used as suggested by developer.
