# Supplementary material for: Chromosome-level assembly of Gymnocypris eckloni genome
Source: Sci Data. 2022 Aug 2;9:464. doi: 10.1038/s41597-022-01595-w (PMC9346132; doi:10.1038/s41597-022-01595-w)
Supplement: Supplementary file 1 — Supplementary information of Chromosome-level assembly of Gymnocypris eckloni genome [file 41597_2022_1595_MOESM1_ESM.pdf]

Supplementary information of Chromosome-level assembly of *Gymnocypris eckloni* genome

Table s1. Statistics of genome size estimation by 17-mer analysis.	Page 2
Table s2. Statistics of the chromosome assemblies using Hi-C data.	Page 2
Table s3. The annotation of repeated sequences in the *G. eckloni* genome using TRF, RepeatMasker and RepeatProteinMask.	Page 3
Table s4. Summary statistics of repeat annotation in *G. eckloni*.	Page 3
Table s5. The number of the annotated non-coding RNA in the *G. eckloni* genome.	Page 3
Table s6. The comparison of the gene models annotated from the homolog species of *G. eckloni*.	Page 4
Table s7 The number of genes with functional classifications in *G. eckloni*.	Page 4
Table s8 Gene families among the 14 species.	Page 5
Fig s1. A picture of *G. eckloni* used in the genome sequencing and assembly.	Page 5

Table s1. Statistics of genome size estimation by 17-mer analysis.

Kmer	*Kmer* number	*Kmer* Depth	Genome size (Mb)	Heterozygosity	Repeat_rate(%)
17	169,021,371,761	181	927.13	1.82%	50.5

Table s2. Statistics of the chromosome assemblies using Hi-C data.

Pseudo-Chromosomes	Sequence IDs	Cluster Numbers	Sequences Lengths
Chr01	Hic_asm_0	170	44,161,858
Chr02	Hic_asm_1	20	25,669,045
Chr03	Hic_asm_2	102	34,715,927
Chr04	Hic_asm_3	125	61,913,669
Chr05	Hic_asm_4	6	28,665,261
Chr06	Hic_asm_5	66	43,543,958
Chr07	Hic_asm_6	154	61,756,641
Chr08	Hic_asm_7	118	47,622,867
Chr09	Hic_asm_8	62	27,783,661
Chr10	Hic_asm_9	69	32,896,108
Chr11	Hic_asm_10	82	21,739,851
Chr12	Hic_asm_11	29	32,200,481
Chr13	Hic_asm_12	42	29,129,546
Chr14	Hic_asm_13	56	28,882,651
Chr15	Hic_asm_14	232	70,525,535
Chr16	Hic_asm_15	195	89,391,071
Chr17	Hic_asm_16	193	16,898,605
Chr18	Hic_asm_17	54	15,913,822
Chr19	Hic_asm_18	68	33,789,651
Chr20	Hic_asm_19	190	46,494,833
Chr21	Hic_asm_20	100	32,874,627
Chr22	Hic_asm_21	96	39,527,105
Chr23	Hic_asm_22	101	39,007,358

Table s3. The annotation of repeated sequences in the *G. eckloni* genome using TRF, RepeatMasker and RepeatProteinMask. Note that the total content was merged and redundancy was eliminated by each method.

Type	Repeat Size(bp)	% of genome
TRF	59,024,407	6.42
RepeatMasker	420,920,632	45.82
RepeatProteinMask	63,100,708	6.87
Total	437,590,862	47.63

Table s4. Summary statistics of repeat annotation in *G. eckloni*. Note that *De novo* + Repbase represent the result of RepeatMasker based on Repbase, RepeatModeler, RepeatScout, and LTR_FINDER; TE proteins meant the result of RepeatProteinMask based on Repbase, and the Combined TEs refer to the combined results of *De novo* + Repbase and TE proteins.

Type	Denovo+Repbase		TE Proteins		Combined TEs	
	Length (bp)	% in genome	Length (bp)	% in genome	Length (bp)	% in genome
DNA	43,441,654	4.73	4,297,129	0.47	44,941,543	4.89
LINE	5,251,877	1.66	30,834,954	3.36	36,383,537	3.96
SINE	2,370,900	0.26	0	0	2,370,900	0.26
LTR	355,341,965	38.68	27,985,849	3.05	356,794,021	38.84
Unknown	11,690,176	1.27	0	0	11,690,176	1.27
Total	420,920,632	45.82	63,100,708	6.87	425,012,609	46.26

Table s5. The number of the annotated non-coding RNA in the *G. eckloni* genome.

<table>
<tr><th colspan="2">Type</th><th>Number</th><th>Average length (bp)</th><th>Total length (bp)</th><th>% of genome</th></tr>
<tr><td colspan="2">miRNA</td><td>1,717</td><td>116.58</td><td>200,168</td><td>0.021789</td></tr>
<tr><td colspan="2">tRNA</td><td>12,157</td><td>75.00</td><td>911,763</td><td>0.099247</td></tr>
<tr><td rowspan="5">rRNA</td><td>rRNA</td><td>1,780</td><td>178.65</td><td>317,991</td><td>0.034614</td></tr>
<tr><td>18S</td><td>207</td><td>437.05</td><td>90,469</td><td>0.009848</td></tr>
<tr><td>28S</td><td>282</td><td>346.36</td><td>97,674</td><td>0.010632</td></tr>
<tr><td>5.8S</td><td>55</td><td>117.29</td><td>6,451</td><td>0.000702</td></tr>
<tr><td>5S</td><td>1,236</td><td>99.84</td><td>123,397</td><td>0.013432</td></tr>
<tr><td rowspan="6">snRNA</td><td>snRNA</td><td>1,152</td><td>133.32</td><td>153,582</td><td>0.016718</td></tr>
<tr><td>CD-box</td><td>172</td><td>120.66</td><td>20,754</td><td>0.002259</td></tr>
<tr><td>HACA-box</td><td>107</td><td>142.94</td><td>15,295</td><td>0.001665</td></tr>
<tr><td>splicing</td><td>826</td><td>132.76</td><td>109,663</td><td>0.011937</td></tr>
<tr><td>scaRNA</td><td>35</td><td>179.94</td><td>6,298</td><td>0.000686</td></tr>
<tr><td>Unknown</td><td>12</td><td>131</td><td>1,572</td><td>0.000171</td></tr>
</table>

Table s6. The comparison of the gene models annotated from the homolog species of *G. eckloni*.

Species	Gene number	Average length(bp)				Exons number per gene
		Transcript	CDS	Exon	Intron	
Gymnocypris eckloni	24,430	16,219.34	1,536.71	173.00	1,862.69	8.88
Cyprinus carpio	47,513	11,443.46	1,214.22	164.14	1,598.93	7.40
Astyanax mexicanus	22,631	20,200.93	1,574.58	162.81	2,148.02	9.67
Danio rerio	26,162	24,859.47	1,620.00	173.68	2,790.70	9.33
Takifugu rubripes	21,944	8,721.04	1,804.84	167.83	709.07	10.75
Ctenopharyngodon idellus	32,659	10,458.83	1,389.10	181.01	1,358.97	7.67
Oryzias latipes	19,146	11,969.78	1,504.25	148.03	1,142.33	10.16
Ictalurus punctatus	22,494	17,637.62	1,753.46	171.17	1,718.35	10.24

Table s7. The number of genes with functional classifications in *G. eckloni*.

Database	Number	Percent (%)
SwissProt	20,432	83.60
NR	23,110	94.60
KEGG	20,593	84.30
InterPro	21,539	88.20
GO	15,281	62.60
Pfam	19,290	79.00
Annotated	23,157	94.80
Unannotated	1,273	5.20
Total	24,430	94.80

Table s8. Gene families among the 14 species.

Species	Genes Number	Single-copy genes	Multiple-copy genes	Unique	Other	Uncluster
G. eckloni	24,428	1,396	4,358	576	1,4782	3,316
T. rubripes	21,391	1,442	4,190	368	14,274	1,117
O. latipes	22,107	1,443	4,286	298	14,858	1,222
C. carpio	23,755	1,299	4,704	338	13,559	3,855
A. mexicanus	18,684	1,580	3,569	231	12,238	1,066
C. idellus	32,659	1,300	4,942	891	17,421	8,105
D. rerio	25,357	1,270	4,957	865	17,167	1,098
I. punctatus	23,599	1,333	4,674	390	15,774	1,428
O. niloticus	28,149	1,347	4,815	1,294	19,416	1,277
H. sapiens	20,155	1,652	3,141	503	14,061	798
M. musculus	22,312	1,681	3,063	1,916	14,730	922
G. gallus	16,628	1,748	2,646	587	10,621	1,026
X. tropicalis	17,204	1,828	2,659	1,007	10,709	1,001
P. marinus	10,127	2,187	1,404	388	4,234	1,914

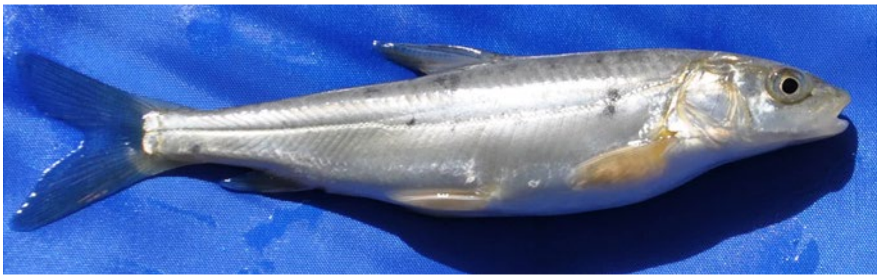

Fig. s1. A picture of *G. eckloni* used in the genome sequencing and assembly.
